# Economics of the Management of Craniospinal Chordoma and Chondrosarcoma and the feasibility of the bundled payment model

**DOI:** 10.1186/s12883-020-01850-w

**Published:** 2020-08-21

**Authors:** Zaid Aljuboori, Beatrice Ugiliweneza, Dengzhi Wang, Norberto Andaluz, Maxwell Boakye, Brian Williams

**Affiliations:** Department of Neurological Surgery, University of Louisville School of Medicine, 220 Abraham Flexner way, Ste.1500, Louisville, KY 40202 USA

**Keywords:** Chordoma, Chondrosarcoma, Bundled payment, Clivus, Spine

## Abstract

**Background:**

The Centers for Medicare and Medicaid Services (CMS) created a new reimbursement model “Bundled Payment for Care Improvement (BPCI)” which reimburses providers a predetermined payment in advance to cover all possible services rendered within a certain time window. Chordoma and Chondrosarcoma are locally aggressive malignant primary bony tumors. Treatment includes surgical resection and radiotherapy with substantial risk for recurrence which necessitates monitoring and further treatment. We assessed the feasibility of the BPCI model in these neurosurgical diseases.

**Methods:**

We selected patients with chordoma/chondrosarcoma from inpatient admission table using the International Classification of Disease, 9th (ICD-9), and 10th (ICD-10) revision codes. We collected the patients’ demographics and insurance type at the index hospitalization. We recorded the following outcomes length of stay, total payment, discharge disposition, and complications for the index hospitalization. For post-discharge, we collected the 30 days and 3/6/12 months inpatient admission, outpatient service, and medication refills. Continuous variables were summarized by means with standard deviations, median with interquartile and full ranges (minimum-maximum); Continuous outcomes were compared by nonparametric Wilcoxson rank-sum test. All tests were 2-sided with a significance level of 0.05. Statistical data analysis was performed in SAS 9.4 (SAS Institute, Inc, Cary, NC).

**Results:**

The population size was 2041 patients which included 1412 patients with cranial (group1), 343 patients with a mobile spine (group 2), and 286 patients with sacrococcygeal (group 3) chordoma and chondrosarcoma. For index hospitalization, the median length of stay (days) was 4, 6, and 7 for groups 1, 2, and 3 respectively (P<.001). The mean payments were ($58,130), ($84,854), and ($82,440), for groups 1, 2, and 3 respectively (P=.02). The complication rates were 30%, 35%, and 43% for groups 1, 2, and 3 respectively (P<.001). Twelve months post-discharge, the hospital readmission rates were 44%, 53%, and 65% for groups 1, 2, and 3, respectively (P<.001). The median payments for this period were ($72,294), ($76,827), and ($101,474), for groups 1, 2, and 3, respectively (P <.001).

**Conclusion:**

The management of craniospinal chordoma and chondrosarcoma is costly and may extend over a prolonged period. The success of BPCI requires a joint effort between insurers and hospitals. Also, it should consider patients’ comorbidities, the complexity of the disease. Finally, the adoptionof quality improvement programs by hospitals can help with cost reduction.

## Background

The continuous rise in healthcare expenditures in the United States represents a dilemma to policy makers, insurers, and patients [1]. Under the current fee-for-service (FFS) system, healthcare providers are reimbursed based on the volume of services performed. This system has been criticized on the basis of rewarding providers for increasing the volume of services, not necessarily the quality of care [2]. For example, there is evidence that colonoscopy for colon cancer screening is being done in increased frequency than recommended [3]. To address that, multiple initiatives have been proposed to reduce the cost and increase the quality of care. The Centers for Medicare and Medicaid Services (CMS) have been experimenting with new reimbursement model the “Bundled Payment for Care Improvement (BPCI)” since 2013. Under this new system, the insurer only pays a pre-specified bundled payment (BP) value in advance to cover all possible services rendered to patients within a specified time window around the treatment, including eventual complications. The payments are calculated using historical financial data [4]. The BPCI initiative involves models 1,2,3 & 4 as a progressive rollout of the plan. Each one of these models has its own definition of the “episode of care”.

In Model 2, the episode includes the inpatient stay in an acute care hospital plus the post-acute care and all related services up to 90 days after hospital discharge.

In contrast to the FFS system, where the insurer reimburses the cost of each test, procedure, hospital stay, etc., including those incurred because of complications and readmissions.

Chordoma and Chondrosarcoma (CC) are relatively rare primary bony tumors. They are slow growing and malignant tumors. Chordoma originates from the remnants of the notochord and it almost always located along the neuroaxis. It can affect areas anywhere from the clivus to the sacrum. Chondrosarcoma is mesenchymal in origin and characterized by formation of cartilage matrix [5, 6]. To date there are no reports in the literature that link the clinical and financial characteristics of the management of chordoma and chondrosarcoma to the potential feasibility of BPCI model.

We report the analyses of data obtained from the MarketScan research database regarding the reimbursements of the management of craniospinal chordoma and chondrosarcoma up to 12 months after index hospitalization. In addition, we discuss the feasibility of the BPCI model considering the results of our analyses.

## Methods

### Data source

We obtained the data from the Truven Health MarketScan Databases with permission to use. MarketScan is a healthcare research database with de-identified medical records of more than 250 million patients, including inpatient, outpatient, and prescription data, diagnoses and procedures, insurer type, and payment information [7]. MarketScan contains multiple tables linked with a unique patient identification number, representing the patients’ trajectories through the healthcare system. So, it can used to study patient’s healthcare utilization longitudinally. For this study, we used the inpatient, outpatient, and medication tables for the years 2000–2015.

### Cohort selection

We selected patients with chordoma/chondrosarcoma from inpatient admission table using the International Classification of Disease, 9th Revision (ICD-9) codes 170.0, 160.2, 143.0, 170.1 and 10th Revision (ICD-10) codes C41.0, C31.0, C03.0, C41.1 for chordoma of skull and face, ICD-9 code 170.2 and ICD-10 code C41.2 for chordoma of vertebral column, ICD-9170.6 ICD-10 C41.4 for chordoma of sacrum/coccyx. For each patient, the first occurring hospitalization was considered the index hospitalization. Pre-diagnosis lookback time was calculated as the difference between and the beginning enrollment date and the date of the index hospitalization admission. Post-diagnosis follow-up time was calculated as the difference between the date of the index hospitalization discharge and end enrollment date. Patients with less than 12 months follow-up time, or with less than 3 months lookback time, or under 18 years old were excluded.

### Patient characteristics

Baseline demographics, insurance type (commercial, Medicaid, Medicare), and comorbidities were summarized at the index hospitalization. Comorbidities were measured with the Elixhauser comorbidity score [8] using ICD-9-CM and ICD-10 codes developed by Quan et al. [9]. The following comorbidities were detected from 3 month before index admission to the index discharge: tobacco use, osteoporosis, hypertension, congestive heart failure (CHF), chronic obstructive pulmonary disease (COPD), myocardial infarction (MI), diabetes, obesity.

### Outcomes

The outcomes of interest were index hospitalization length of stay (LOS), total payment, discharge disposition, and complications. For post-discharge healthcare use and payment, we collected the 30 days, 3 months, 6 months, and 12 months inpatient admission, outpatient services, and medication refills. The bundle payments were calculated as the payments accumulated from the index hospitalization admission to 90 days of post discharge date. All payments were inflated to 2016 US dollars using the medical component of the consumer price index accessible through United States Bureau of Labor Statistics website [7, 10]. Complications were flagged by the presence of the following events on the index complication claim: renal, cardiac, nervous system complication, cerebrovascular disease deep vein thrombosis or pulmonary embolism, pulmonary, infection, pneumonia, and wound.

### Statistical analysis

Continuous variables were summarized by means with standard deviations, median with interquartile and full ranges (minimum - maximum); categorical variables were summarized by counts and percentages. Continuous outcomes were compared by nonparametric Wilcoxson rank sum test; categorical outcomes were compared among groups by Chi-squared test. Adjusted group comparisons of healthcare use and payment were obtained from linear contrasts of multivariable regression models which includes covariates age, gender, Elixhauser index and insurance, in addition to group. Odds ratios were obtained for the demographic variables on the 90 days bundle payment for each group from multilinear regression. All tests were 2-sided with a significance level of 0.05. Statistical data analysis was performed in SAS 9.4 (SAS Institute, Inc., Cary, NC).

## Results

### Demographics

**Fig. 1:**
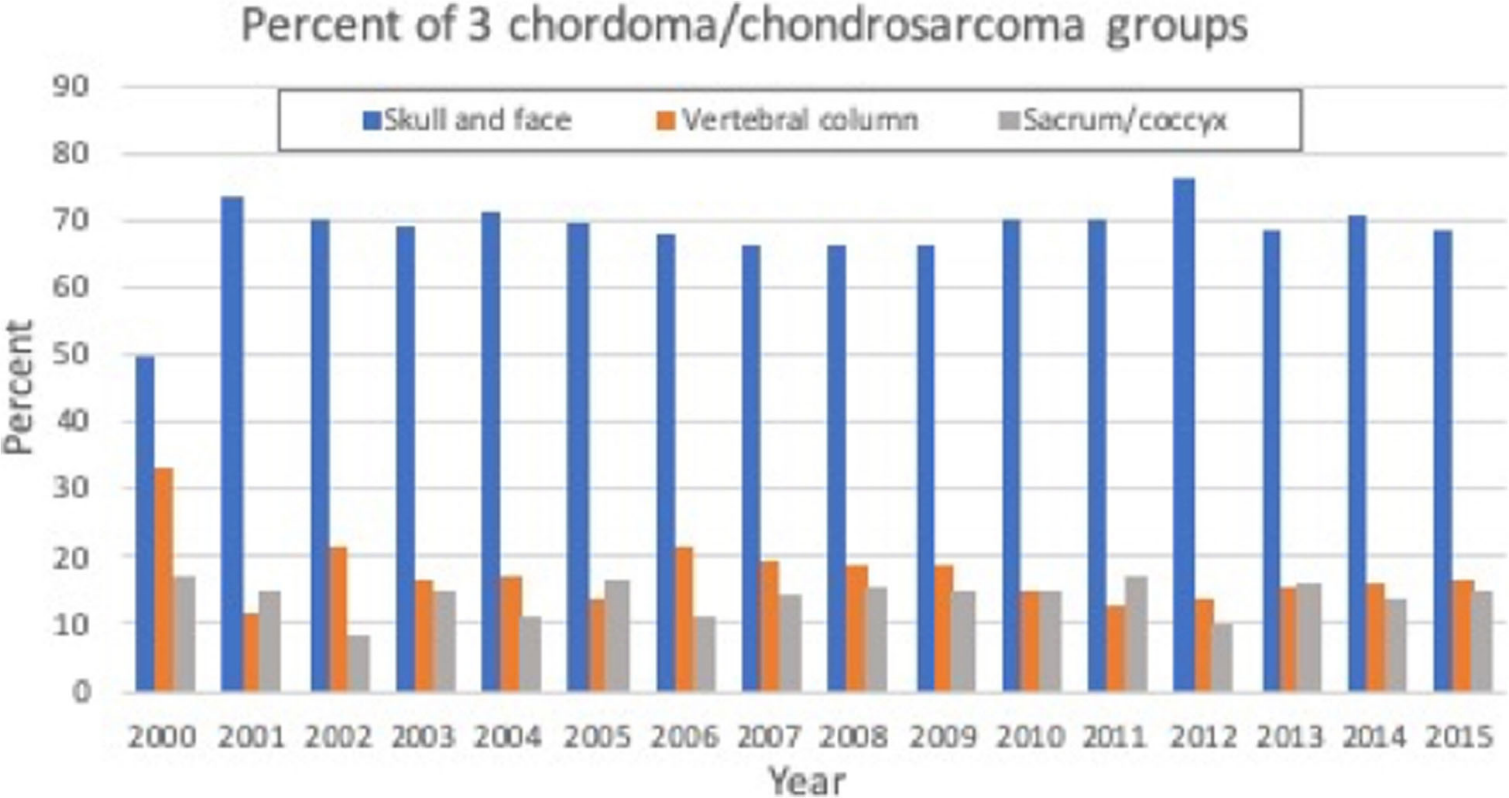
A bar graph shows the percentage of individual group of chordoma and chondrosarcoma (cranial, mobile spine, and sacrococcygeal) over time (2000–2016)

A total of 2041 patients were included. Of those patients 69% (*N* = 1214) had cranial (group 1), 16.9% (*N* = 297) had mobile spine (group 2), and 14% (*N* = 246) had sacrococcygeal (group 3) chordoma and chondrosarcoma [Fig. 1]. The mean age was 57.4, 49.5, and 47.9 years for the cranial, mobile spine, and sacrococcygeal groups, respectively (*P* < .001). Females represented 43%, 48%, and 44% of the cranial, mobile spine, and sacrococcygeal groups, respectively. Of the cranial group, 60%, 12%, and 28% had commercial, Medicaid, and Medicare insurance, respectively. Of the mobile spine group, 68%, 14%, and 17% had commercial, Medicaid, and Medicare insurance, respectively. Of the sacrococcygeal group, 73%, 12%, and 14% had commercial, Medicaid, and Medicare insurance, respectively. See [Tables 1 and 2] for additional details.

**Table 1:** Demographics stratified by group

	Chordoma / chondrosarcoma				
Variable	**Group 1**	**Group 2**	**Group 3**	*p*-value	Combined cohort
	Skull and face	Vertebral column	Sacrum/ coccyx		
Total *N* = 2041	*n* = 1214 (69.1%)	*n* = 297 (16.9%)	*n* = 246 (14%)		*N* = 1757
**Demographics**					
Age				**<.0001**	
Mean (SD)	57.4 (15.8)	49.5 (17)	47.9 (17.2)		54.8 (16.7)
Median (IQR)	58 (49, 69)	52 (37, 62)	49 (35, 61)		56 (45, 65)
Range, min-max)	18–96	18–88	18–89		18–96
Gender: female, n (%)	519 (42.7%)	144 (48.4%)	109 (44.3%)	0.2018	772 (43.9%)
Insurance				**<.0001**	
Commercial, n (%)	726 (59.8%)	203 (68.3%)	181 (73.5%)		1110 (63.1%)
Medicaid, n (%)	144 (11.8%)	42 (14.1%)	30 (12.2%)		216 (12.2%)
Medicare, n (%)	344 (28.3%)	52 (17.5%)	35 (14.2%)		431 (24.5%)
Elixhauser index				0.054	
1, n (%)	415 (34.1%)	85 (28.6%)	72 (29.2%)	0.054	572 (32.5%)
2, n (%)	468 (38.5%)	123 (41.4%)	87 (35.3%)		678 (38.5%)
3+, n (%)	1214 (17%)	297 (15.3%)	246 (14.1%)		1757 (28%)

**Table 2:** Comorbidities frequency stratified by group

Comorbidity	All patients (*N* = 2041)			
	Group 1	Group 2	Group 3	*p*-value
	Skull and face	Vertebral column	Sacrum/ coccyx	
	*n* = 1214 (69.1%)	*n* = 297 (16.9%)	*n* = 246 (14%)	
Tobacco use, n (%)	219 (18%)	34 (11%)	40 (16%)	**0.0235**
Osteoporosis, n (%)	25 (2%)	13 (4%)	1 (0.4%)	**0.006**
Hypertension, n (%)	528 (43.4%)	104 (35%)	77 (31%)	**0.0002**
CHF, n (%)	45 (3.7%)	5 (1.68%)	9 (3.6%)	0.2134
COPD, n (%)	180 (14.8%)	35 (11.78%)	22 (9%)	**0.0308**
MI, n (%)	120 (9.8%)	25 (8.42%)	24 (9.7%)	0.7419
Diabetes, n (%)	169 (13.9%)	36 (12.12%)	25 (10%)	0.2423
Obesity, n (%)	66 (5.4%)	20 (6.73%)	21 (8.5%)	0.1575
At least one of the above, n (%)	807 (66.4%)	172 (57.9%)	133 (54%)	**0.0001**

### Index hospitalization and 30 days post discharge outcomes

Index hospitalization, the median length of stay (days) was 4, 6, and 7 for groups 1, 2, and 3 respectively (*P* < .001). The mean payments were ($58,130), ($84,854), and ($82,440), for groups 1, 2, and 3 respectively (*P* = .02). The complication rates were 30%, 35%, and 43% for groups 1, 2, and 3 respectively (*P* < .001).

Thirty days post discharge, the emergency department admissions were 10%, 12%, and 18% for groups 1, 2, and 3, respectively (*P* = .001). The hospital readmissions were 10%, 23%, and 30% for groups 1, 2, and 3, respectively (*p* < .001). The complication rates were 18%, 24%, 30% for groups 1, 2, and 3, respectively (*P* < .001) [Table 3].

**Table 3:** Outcome comparison among groups (12 months follow up)

	Chordoma / chondrosarcoma				
	Group 1	Group 2	Group 3	p-value	Combined cohort
Variable	Skull and face	Vertebral column	Sacrum/ coccyx		
Total *N* = 2041	*n* = 1214 (69.1%)	*n* = 297 (16.9%)	*n* = 246 (14%)		*N* = 1757
**Index hospitalization outcomes**					
Length of stay, median (IQR)	4 (2, 8)	6 (3, 10)	7 (3, 13)	**<.0001**	5 (2, 9)
Prolonged LOS (> Q3 + 1.5*IQR), n (%)	54 (4.4%)	23 (7.7%)	10 (4%)	0.0501	87 (4.9%)
Payment, median (IQR)	35,490 (19,358, 70,885)	40,476 (17,262, 111,365)	44,038 (21,954, 93,871)	**0.0024**	37,575 (19,307, 81,444)
Discharge home, n (%)	1087 (89.5%)	222 (74.7%)	179 (72.7%)	**<.0001**	1488 (84.6%)
Complications, n (%)	368 (30.3%)	105 (35.3%)	107 (43.5%)	**0.0002**	580 (33%)
**Post discharge outcomes, 30 days**					
ER admission, n (%)	118 (9.7%)	35 (11.7%)	44 (17.8%)	**0.001**	197 (11.2%)
Hospital re-admission, n (%)	126 (10.3%)	69 (23.2%)	75 (30.4%)	**<.0001**	270 (15.3%)
Complications, n (%)	214 (17.6%)	72 (24.2%)	73 (29.6%)	**<.0001**	359 (20.4%)
**Post discharge outcomes, 3 months**					
Hospital admissions					
Admitted, n (%)	251 (20.6%)	114 (38.3%)	110 (44.7%)	**<.0001**	475 (27%)
# readmissions, median (IQR)	0 (0, 0)	0 (0, 1)	0 (0, 2)	**<.0001**	0 (0, 1)
Payments, median (IQR), for Admitted	24,116 (11,216, 56,575)	40,277 (17,418, 90,354)	42,242 (24,991, 122,030)	**<.0001**	33,756 (14,227, 75,308)
Outpatient services					
# services, median (IQR)	67 (24, 126)	58 (29, 106)	64 (29, 122)	0.4646	65 (26, 122)
Payments, median (IQR)	20,522 (4201, 55,275)	14,050 (4283, 36,340)	13,280 (4199, 33,430)	**0.0026**	17,476 (4203, 48,565)
Medication refills					
# refills, median (IQR)	8 (2, 16)	8 (2, 17)	10 (1, 17)	0.64	8 (2, 16)
Payments, median (IQR)	405 (24, 1149)	389 (0, 1977)	676 (11, 2243)	**0.0072**	417 (19, 1355)
Overall payments, median (IQR)	27,590 (6011, 68,534)	25,968 (7250, 76,901)	35,819 (8071, 87,857)	**0.0446**	28,292 (6591, 71,439)
**Post discharge outcomes, 6 months**					
Hospital admissions					
Admitted, n (%)	368 (30.3%)	134 (45.1%)	137 (55.6%)	**<.0001**	639 (36.3%)
# readmissions, median (IQR)	0 (0, 1)	0 (0, 1)	1 (0, 2)	**<.0001**	0 (0, 1)
Payments, median (IQR), for Admitted	26,702 (10,917, 64,297)	46,796 (17,418, 119,961)	51,364 (24,491, 145,701)	**<.0001**	363,19 (13,396, 84,076)
Outpatient services					
# services, median (IQR)	113 (50, 189)	105 (53, 190)	119 (59, 202)	0.1221	112 (52, 192)
Payments, median (IQR)	35,221 (10,151, 79,143)	25,831 (9187, 69,684)	26,880 (8842, 65,760)	0.0661	31,478 (9713, 76,301)
Medication refills					
# refills, median (IQR)	15 (5, 27)	13 (2, 30)	19 (4, 34)	0.1484	15 (4, 28)
Payments, median (IQR)	804 (75, 2198)	759 (13, 3411)	1458 (86, 4692)	**0.0024**	869 (60, 2587)
Overall payments, median (IQR)	48,508 (15,360, 99,994)	49,425 (13,997, 129,738)	60,853 (17,459, 158,391)	**0.0063**	51,088 (15,378, 109,462)
**Post discharge outcomes, 12 months**					
Hospital admissions					
Admitted, n (%)	538 (44.3%)	157 (52.8%)	159 (64.6%)	**<.0001**	854 (48.6%)
# readmissions, median (IQR)	0 (0, 1)	1 (0, 2)	1 (0, 4)	**<.0001**	0 (0, 1)
Payments, median (IQR), for Admitted	30,079 (11,720, 70,475)	53,276 (16,831, 128,733)	71,960 (27,585, 211,350)	**<.0001**	41,437 (14,960, 104,371)
Outpatient services					
# services, median (IQR)	166 (90, 276)	165 (92, 285)	211 (111, 328)	**0.0002**	173 (93, 287)
Payments, median (IQR)	51,375 (18,632, 105,100)	40,646 (16,729, 113,428)	48,804 (20,146, 100,134)	0.5921	49,700 (17,715, 105,100)
Medication refills					
# refills, median (IQR)	26 (8, 49)	22 (5, 51)	33 (10, 59)	**0.0389**	26 (8, 51)
Payments, median (IQR)	1477 (189, 4113)	1470 (70, 7827)	2499 (203, 8708)	**0.0005**	1573 (161, 5102)
Overall payments, median (IQR)	72,294 (28,832, 146,914)	76,827 (28,237, 193,631)	101,475 (39,062, 244,231)	**<.0001**	77,225 (29,520, 163,677)

### Three- and twelve-months post discharge outcomes

Three months post-discharge, the hospital readmission rates were 21%, 38%, and 45% for groups 1, 2, and 3, respectively (*P* < .001). There was no difference in the number of outpatient services and medications refill among the groups. The overall median payments for this period were ($27,590), ($25,968), and ($35,819), for groups 1, 2, and 3, respectively (*P* = .04) [Table 3].

**Fig. 2:**
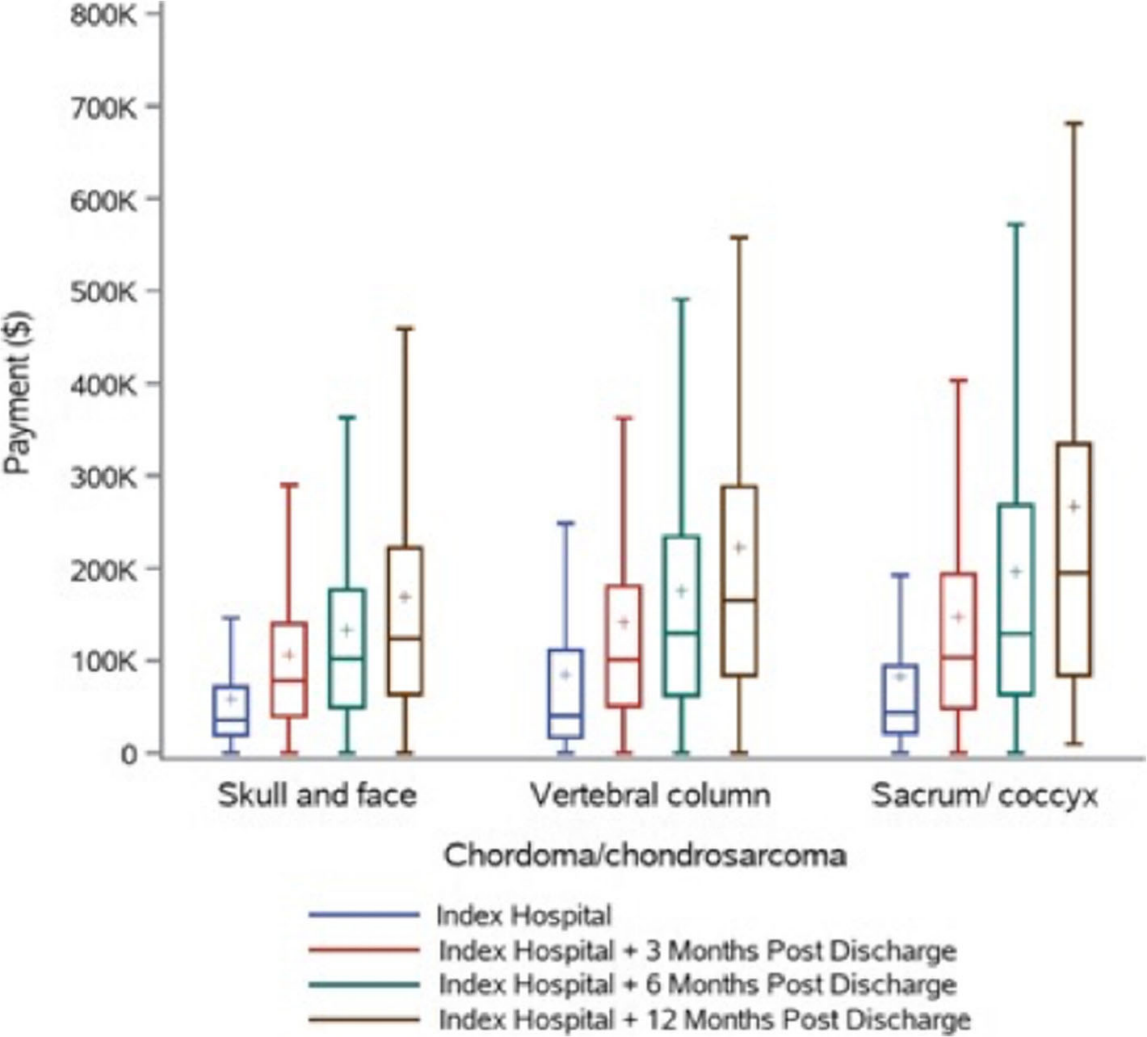
A box and whisker graph show the cumulative payments for managing chordoma/ chondrosarcoma over 12 months divided by groups

**Fig. 3:**
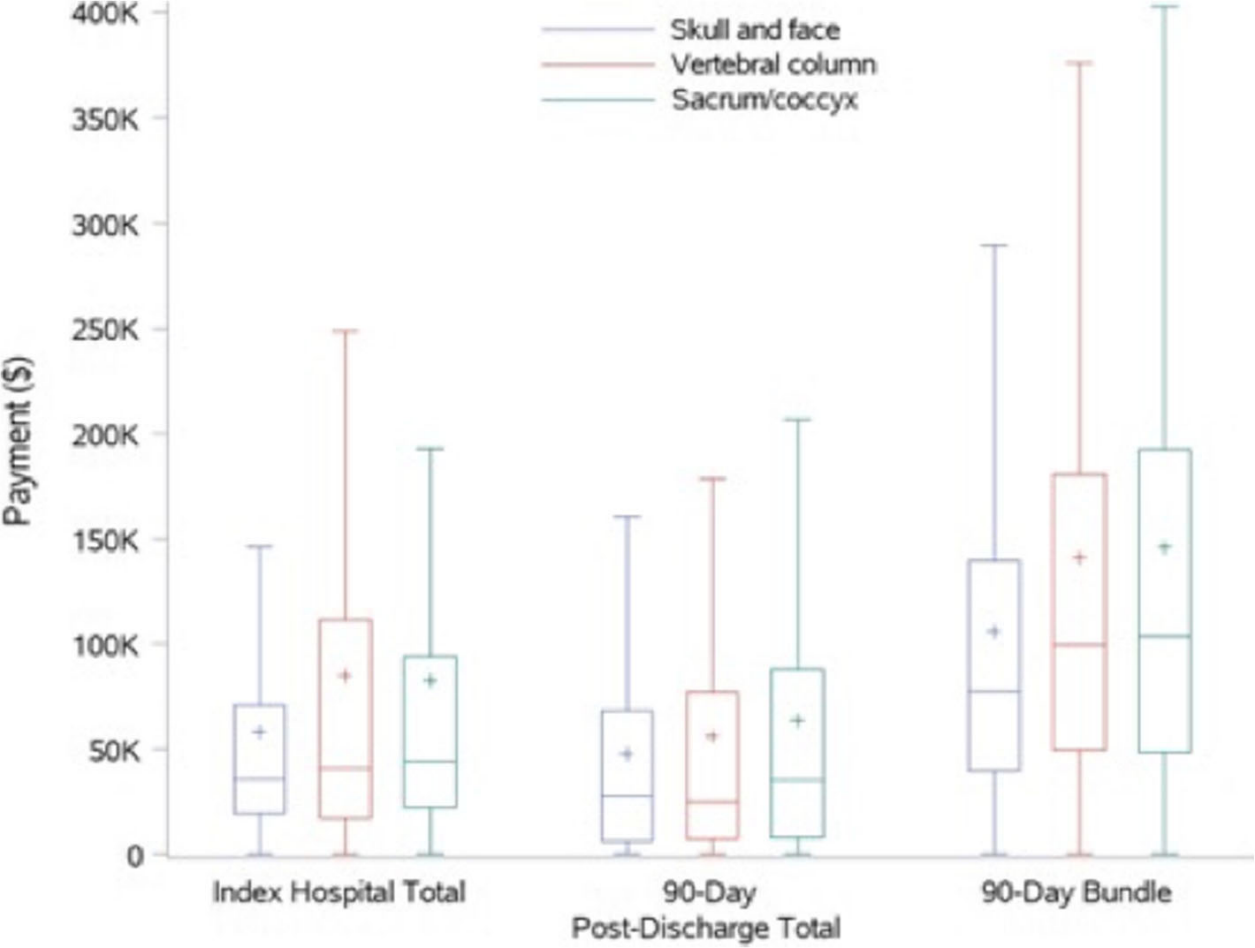
A box and whisker graph show the payments for index hospitalization, 90 days post discharge, and combined “bundled” payments for managing chordoma/ chondrosarcoma divided by groups

Twelve months post-discharge, the hospital readmission rates were 44%, 53%, and 65% for groups 1, 2, and 3, respectively (*P* < .001). The median number of outpatient services rendered was 166, 165, and 211, for groups 1, 2, and 3, respectively (*P* < .001). The overall median payments for this period were ($72,294), ($76,827), and ($101,474), for groups 1, 2, and 3, respectively (*P* < .001) [Table 3], [Fig. 2.]. For the bundled payment for the index hospitalization and 90 days post discharge see [Table 4], [Fig. 3].

**Table 4:** Bundled payment, 3 months period

Variables	All patients (*N* = 2041)			
	Group 1	Group 2	Group 3	*p*-value
	Skull and face	Vertebral column	Sacrum/ coccyx	
	*n* = 1214 (69.1%)	*n* = 297 (16.9%)	*n* = 246 (14%)	
**90-day bundle**				**<.0001**
Mean (SD)	105,765 (101523)	140,898 (138743)	145,961 (143723)	
Median (Q1, Q3)	77,598 (39,513, 139,580)	99,323 (49,428, 180,441)	103,309 (48,361, 192,421)	
Min-Max	0–1,130,642	0–898,360	206–1,000,831	
**Index hospitalization**				
**Total payment**				**0.0024**
Mean (SD)	58,130 (69865)	84,854 (109058)	82,440 (109702)	
Median (Q1, Q3)	35,490 (19,358, 70,885)	40,476 (17,262, 111,365)	44,038 (21,954, 93,871)	
Min-Max	0–732,975	0–772,519	1–940,505	
**Physician payment**				0.0733
Mean (SD)	6051 (9728)	8113 (16816)	6076 (13187)	
Median (Q1, Q3)	3255 (366, 7275)	2750 (420, 8418)	1887 (448, 5614)	
Min-Max	0–118,600	0–162,038	0–119,125	
**Hospital payment**				**0.0018**
Mean (SD)	35,753 (52372)	50,524 (76451)	49,396 (71568)	
Median (Q1, Q3)	19,602 (10,146, 40,830)	21,008 (8204, 62,631)	25,444 (11,934, 60,559)	
Min-Max	0–603,034	0–572,341	0–720,467	
**90-day post-discharge**				
**Total payment**				**0.0484**
Mean (SD)	47,636 (65458)	56,044 (77843)	63,521 (76930)	
Median (Q1, Q3)	27,404 (5914, 68,092)	24,923 (7048, 76,730)	35,292 (7983, 87,857)	
Min-Max	0–1,090,012	0–525,663	0–360,038	
**Re-admission payment**				**<.0001**
Mean (SD)	9900 (42208)	26,549 (60722)	35,003 (62071)	
Median (Q1, Q3)	0 (0, 0)	0 (0, 22,266)	0 (0, 37,832)	
Min-Max	0–1,074,424	0–484,967	0–279,163	
**Outpatient services payment**				**0.002**
Mean (SD)	36,637 (49919)	27,202 (39092)	25,958 (36611)	
Median (Q1, Q3)	20,027 (4129, 54,781)	13,777 (4092, 33,597)	12,775 (4146, 32,938)	
Min-Max	0–793,307	0–274,015	0–254,750	
**Medication payment**				**0.0121**
Mean (SD)	1099 (2842)	2293 (5404)	2560 (5012)	
Median (Q1, Q3)	394 (23, 1123)	389 (0, 1977)	631 (9, 2222)	
Min-Max	0–61,580	0–35,502	0–30,726

### Adjusted comparison among groups

Using the cranial group (1) as a reference, the index hospitalization of the combined spinal group (groups 2, and 3) had increased length of stay (RR1.2, 1.6, *P* < .001), a higher complications rate (RR 1.1, 1.8, *P* < .001), and decreased rate of discharge to home (0RR0.3, 0.25, *P* < .001). Thirty days post-discharge, the combined spinal group (groups 2 and 3) had a higher ED admission (OR 1.08, 1.7, *P* = .01), hospital readmission (OR 2.3, 3.1, *P* < .001), and complications rate (OR 1.5, 2.4, *P* < .001). Three months post-discharge, the combined spinal group (groups 2, and 3) had a higher hospital admission (OR 2, 2.6, *P* < .001), and a decreased use of outpatient services (RR 0.8, 0.9, *P* < .001). The twelve months post-discharge, the combined spinal group (groups 2, and 3) had a higher hospital readmission (OR 1.2, 2.1, *P* < .001), higher medication refill (RR 1.03, 1.1, *P* < .001), and a had higher overall payment (RR 1.02, 1.2, *P* = .02).

### Ninety days multivariate analysis

Increased age by 10 years increment was associated with a decreased payment for groups 1, 2, and 3, (OR 0.9, 0.89, and 0.88). Medicaid insurance was associated with a decreased payment for groups 1, 2, and 3 (OR 0.5, 0.3, and 0.47) in comparison to commercial insurance. Medicare was associated with a decreased payment only for group 1 (OR 0.77). EI of 2 was associated with a higher payment for groups 1 and 2 (OR 1.1 and 1.5), while EI of 3 was associated with a higher payment for groups 1, 2, and 3 (OR 1.4, 1.5, 1.4) [Table 5].

**Table 5:** Odds Ratio and 95% CI from Multivariable analysis for 90 days bundle payment

		All patients (N = 2041)		
		Group 1	Group 2	Group 3
		Skull and face	Vertebral column	Sacrum/ coccyx
**Cofactor**	**Category**	*n* = 1214 (69.1%)	*n* = 297 (16.9%)	*n* = 246 (14%)
**Age**	+ 10 year	0.9 (0.888, 0.97)	0.8 (0.827, 0.974)	0.8 (0.809, 0.966)
**Gender**	Female vs Male	0.9 (0.847, 1.051)	1.1 (0.928, 1.444)	0.9 (0.761, 1.281)
**Insurance**	Medicaid vs Commercial	0.5 (0.428, 0.692)	0.3 (0.174, 0.64)	0.4 (0.267, 0.838)
**type**	Medicare vs Commercial	0.7 (0.646, 0.93)	0.8 (0.534, 1.297)	0.5 (0.27, 1.2)
**Elixhauser**	Score 2 vs 1	1.1 (1.008, 1.363)	1.4 (1.084, 1.992)	1.3 (0.957, 1.79)
**score**	Score 3 vs 1	1.4 (1.273, 1.696)	1.5 (1.149, 2.078)	1.4 (1.063, 1.92)

## Discussion

Chordoma and chondrosarcoma of the craniospinal axis are challenging neurosurgical conditions [6]. The management paradigm includes maximum safe resection and radiotherapy [11]. The treatment of these conditions can be costly because of the multitude of services required. For example, the treatment of spinal chordoma generally requires a complex spine procedure such as en-bloc surgical excision and multilevel instrumented fusion [5, 12]. This generally is associated with increased length of stay,need for rehabilitation, complications, the risk for emergency department visits, hospital readmission, need for pain prescription refills, and cost [5, 11, 13]. Both diseases have elevated risk for recurrence (~ 57% for cranial and 27% for spinal disease) which may necessitate further treatment [12, 14–16]. These factors make these two conditions valuable to explore the cost and patterns of expenditure over time and the feasibility of adopting the BPCI model for reimbursement.

Notably, the current bundles, as indicated on the CMS website include specific clinical situations such as acute Myocardial Infarction, Sepsis, CABG, etc., which are frequent and well-defined clinical entities. The current list of bundles does not mention any skull base tumors or malignancies, probably because of the difficulty to accurately define them as individual clinical entities. The choice of chordomas and chondrosarcomas in this study may represent a limitation, given both the rarity of those tumors and the variable outcomes of these diagnoses. But it can be a strength since the BPCI will probably be the standard method for payment for all diseases.

For index hospitalization, our analyses showed that spinal CC patients had increased LOS and complications rate, which was associated with higher median payment compared to the cranial group. This could be related to surgical pain, surgical drains, and the need for in-hospital rehabilitation. These factors should be considered when estimating the bundled payments for CC patients. About 90% of the index hospitalization payments were hospital payments with only 10% for physicians’ payment. This magnifies the effects of LOS and services rendered during hospitalization on cost.

Ninety days post-discharge, the readmission rates were higher for spinal CC (group 2, 38% and group 3, 45%) compared to cranial CC (group1, 21%) and were associated with higher median payments ($40,227 and $42,242 for groups 2 and 3 vs $24,116 for group 1). The higher readmission rates for the spinal CC patients could have been due to pain or wound problems when compared to the cranial group [17]. Fry et al. reported a 90 days readmission rate of 25% after elective craniotomy for a mass lesion. Seizures, sepsis, wound complications, pneumonia, and postoperative infections were the most common causes for readmission [18], while Lau et al, reported a 90 days readmission rate of 13% and 20% after surgery for spinal chordoma and chondrosarcoma, respectively. They also reported that wound infection, tumor recurrence requiring decompression, postoperative pain, and proximal junctional kyphosis requiring revision procedure were the most common causes for readmission [19]. The difference in readmission rates between this study and Lau et al could have been due to the smaller sample size in their study (23 chordomas, 10 chondrosarcomas).

Over time, all groups showed a downward trend in hospital readmissions, outpatient service utilization, prescription refills, and median payments. For cranial CC, during the first 6 months post-index hospitalization, the readmission rate was 30% (21% for initial 90 days), outpatient services were 113, and the overall median payment was $48,508 ($24,116 for initial 90 days). For the second 6 months post initial discharge, there was a decline in readmission rate 14%, outpatient services 53, and the median payment of $23,786.For spinal CC, during the first 6 months post-index hospitalization, the readmission rate was 45% mobile spine vs 55% sacrum (38% vs 45% for initial 90 days), outpatient services were 105 mobile spine vs 119, and the median payment was $49,425 mobile spine vs $60,853 sacrum ($40,227 vs $42,242 for initial 90 days). During the second 6 months there was a decline in readmission rate 10% mobile spine vs 9% sacrum, outpatient services 60 mobile spine vs 92 sacrum, and median payment $22,869 mobile spine vs $40,622. These trends indicated less utilization of healthcare services and cost reduction which might have been because of healing, reduction in postoperative pain, and improved functional status with rehabilitation. Hospital readmission was the main factor for the costs incurred during the first 12 months post-discharge and to a lesser extent outpatient services utilization. Besides, most of the expenses were during the first 6 months post-index hospitalization.

There was significant variability in payments based on insurance type, Medicaid was associated with increased odds for smaller payment for all groups, while Medicare was associated with increased odds for smaller payment only for cranial CC when compared to commercial insurers. Also, higher EI value (multiple comorbidities) was associated with increased odds for larger payments for all groups, which emphasizes the effect of patients’ comorbidities on the cost of care. Therefore, using a tool like the CMS Human Health Services (HHS) Hierarchical Condition Category (HCC) risk adjustment model can be helpful. This model uses patients’ demographic data and coded diagnoses to produce a risk score that will help with financial estimation [20]. Turcot et al. recently published a report where they tested this model on patients that underwent different spinal surgical interventions. They found that there was a significant association between the HCC score and readmission rates, length of stay, need for reoperation, and cost [21].

The success of BPCI requires a joint effort between insurers and hospitals/providers. Our analysis showed that complex neurosurgical conditions like craniospinal CC have increased risk for complications, readmissions, and the need for outpatient services. Therefore, a specific BPCI model might be needed to balance the cost and quality of care. We suggest that BPCI should consider bundling the payments for the index hospitalization and the anticipated services during the first six months after initial discharge. It also should consider patients’ comorbidities and the variability in treatment regimens like the use of experimental and off label treatments. Also, it's crucial to streamline and minimize the variability in reimbursements between Medicare/ Medicaid and commercial insurers. Besides, hospitals and providers should consider measures that can improve outcomes and decrease costs. Adoption of programs like Enhanced Recovery After Surgery (ERAS) or Enhanced Perioperative Care (EPOC) which were designed to decrease the length of stay, complications rate, and readmissions can be valuable [22, 23]. Finally, it’s well-documented that treatment at a center with high case volume is associated with better outcomes and lower complications rate, which in turn leads to lower cost [24–26]. Therefore, it might be important for the BPCI to stipulate that certain rare and complex neurosurgical conditions should be managed at centers of excellence.

## Limitations

The limitations of our study and the MarketScan database should be recognized in light of the results. The database combined data for both chordoma and chondrosarcoma because both conditions were coded using the same ICD-9/10 codes. The database does not include data on various surgical techniques, the extent of resection, and the exact anatomical location of the tumor. Besides, the MarketScan database has information on complications, readmission, and outpatient services. It doesn't include information regarding details such as causes for readmission, complications, radiotherapy use, and type of radiotherapy used. Also, there are no data on recurrence and the need for re-resection. Patients paid out of pocket for treatment were not represented in this report. Finally, local treatment biases might have influenced the data retrieved from the MarketScan database. Therefore, it may not be entirely representative of the national chordoma and chondrosarcoma population. Notably, our data will probably include some cases of osteosarcoma and Ewing’s sarcoma due to coding limitations. Nevertheless, because of the size of the patient population,the data will have value despite these limitations. Also, primary osteosarcomas of the skull and skull baseare quite rare, comprising < 2% of all skull tumors. Besides, primary osteosarcoma of the spine is rare, accounting for 3–5% of all osteosarcomas [27, 28]. Ewing’s sarcoma occurs mostly in children who were excluded from the study population. Also, Ewing’s sarcoma of the spine is rare, represents approximately 0.9% of all cases [29, 30].

## Conclusion

The fee-for-service system reimbursement is based on the volume of services performed. According to the Centers for Medicare and Medicaid Services (CMS) it is contributing to the rise in healthcare expenditures. The BPCI model aims to improve outcomes and decrease cost. Chordoma and Chondrosarcoma are malignant bony tumors that requires complex surgical intervention, possible radiotherapy, and a battery of outpatient services. Therefore, they are valuable to evaluate the feasibility of BPCI. To succeed, the BPCI should consider patients’ comorbidities, disease complexity, and risk for complications. Also, hospitals should take measures to reduce cost through applying quality improvement programs and restrict unnecessary services. The BPCI model can be feasible for the management of craniospinal chordoma and chondrosarcoma if stratified by location and covered the services provided during the index hospitalization and the 6 months post discharge.

## Data Availability

The data supporting our findings are presented in the tables of the manuscript.

## Abbreviations

CMS: Centers for Medicare and Medicaid Services
BPCI: Bundled Payment for Care Improvement
FFS: Fee-for-service
BP: Bundled payment
CHF: Congestive heart failure
COPD: Chronic obstructive pulmonary disease
MI: Myocardial infarction
ICD-9: International Classification of Disease, 9th Revision
HHS: Human Health Services
HCC: Hierarchical Condition Category
ERAS: Enhanced Recovery After Surgery
EPOC: Enhanced Perioperative Care
CC: Chordoma / chondrosarcoma
CABG: Coronary artery bypass graft.
